# Allele combinations of maturity genes *E1*-*E4* affect adaptation of soybean to diverse geographic regions and farming systems in China

**DOI:** 10.1371/journal.pone.0235397

**Published:** 2020-07-06

**Authors:** Luping Liu, Wenwen Song, Liwei Wang, Xuegang Sun, Yanping Qi, Tingting Wu, Shi Sun, Bingjun Jiang, Cunxiang Wu, Wensheng Hou, Zhongfu Ni, Tianfu Han

**Affiliations:** 1 Ministry of Agriculture and Rural Affairs Key Laboratory of Soybean Biology (Beijing), Institute of Crop Sciences, Chinese Academy of Agricultural Sciences, Beijing, China; 2 State Key Laboratory of Agrobiotechnology, Key Laboratory of Crop Heterosis and Utilization (MOE), College of Agronomy and Biotechnology, China Agricultural University, Beijing, China; University of Missouri Columbia, UNITED STATES

## Abstract

Appropriate flowering and maturity time are important for soybean production. Four maturity genes *E1*, *E2*, *E3* and *E4* have been molecularly identified and found to play major roles in the control of flowering and maturity of soybean. Here, to further investigate the effect of different allele combinations of *E1*-*E4*, we performed Kompetitive Allele Specific PCR (KASP) assays based on single nucleotide polymorphisms (SNPs) at these four *E* loci, and genotyped *E1*-*E4* genes across 308 Chinese cultivars with a wide range of maturity groups. In total, twenty-one allele combinations for *E1*-*E4* genes were identified across these Chinese cultivars. Various combinations of mutations at four *E* loci gave rise to the diversity of flowering and maturity time, which were associated with the adaptation of soybean cultivars to diverse geographic regions and farming systems. In particular, the cultivars with mutations at all four *E* loci reached flowering and maturity very early, and adapted to high-latitude cold regions. The allele combinations *e1-as*/*e2-ns*/*e3-tr*/*E4*, *E1*/*e2-ns*/*E3*/*E4* and *E1*/*E2*/*E3*/*E4* played important roles in the Northeast China, Huang-Huai-Hai (HHH) Rivers Valley and South China regions, respectively. Notably, *E1* and *E2*, especially *E2*, affected flowering and maturity time of soybean significantly. Our study will be beneficial for germplasm evaluation, cultivar improvement and regionalization of cultivation in soybean production.

## Introduction

Soybean [*Glycine max* (L.) Merrill], a short-day crop, is sensitive to photoperiod. Appropriate flowering and maturity time in suitable planting areas are critical for soybean production. Soybean cultivars from different latitude environments possess different photoperiod sensitivity, which is an essential characteristic to flower and mature at the right time. For convenience of cultivation regionalization, a maturity group (MG) system has been well developed and commonly used to estimate the range of ecological adaptation in North America [1,2]. In China, extensive studies have categorized soybean accessions into different MGs based on environment and planting patterns [3–5]. These years, identification of DNA markers increased the efficiency of marker-assisted selection (MAS) for complex traits including flowering and maturity time of soybean [3,4,6,7]. Thus, it is important to deeply understand the effect of allelic variations of maturity genes on adaptation of soybean cultivars to diverse geographic regions and farming systems.

The flowering and maturity of soybean is genetically controlled by multiple loci known as the *E* series and *J* (long Juvenility gene), among which *E1*-*E4*, *E9* and *J* have been cloned and partly characterized [8–14]. Four loci of *E1*, *E2*, *E3* and *E4* play important roles in regulating flowering and maturity, and some natural variations of these genes have been identified across abundant soybean germplasm [8–11,15–18]. *E1* (Glyma.06g207800), a legume-specific gene, represses flowering of soybean under long-day (LD) conditions [8]. *E2* (*GmGIa*, Glyma.10g221500), an ortholog of Arabidopsis *GIGANTEA*, is a flowering repressor in soybean [9]. *E3* (*GmPHYA3*, Glyma.19g224200) and *E4* (*GmPHYA2*, Glyma.20g090000) suppress soybean flowering by responding to different light qualities under LDs [10,11,19,20]. For the *E1* locus, the alleles *e1-fs* (frame shift), *e1-as* (amino acid substitution), *e1-b3a* (mutation in B3 domain), *e1-re* (retrotransposon insertion) and *e1-p* (allele from cultivar ‘Peking’) have single nucleotide polymorphisms (SNPs) or insertion-deletions (InDels) in the coding sequence or 5’ upstream, and *e1-nl* (null) allele has a 130 kb deletion which includes the entire *E1* gene [8,15,18]. For the *E2* locus, the recessive allele *e2* or *e2-ns* (nonsense mutation) has a single-base substitution in the 10th exon which results in a premature stop codon [9,18]. For the *E3* locus, *e3-ns* (nonsense mutation), *e3-fs* (frame shift) and *e3-Mo* (allele from cultivar ‘Moshidou Gong 503’) have SNPs in the exons, *e3-tr* (large deletion after the third exon) and *E3-Ha* (allele from cultivar ‘Harosoy’) have a 13.3 kb deletion (including exon 4) and a 2.6 kb insertion after the third exon respectively comparing to *E3-Mi* (allele from cultivar ‘Misuzudaizu’) [10,16,18]. The *e4-oto* (allele from cultivar ‘Otomewase’), *e4-tsu* (allele from cultivar ‘Tsukue-4’), *e4-kam* (allele from cultivar ‘Kamaishi-17’), and *e4-kes* (allele from cultivar ‘Keshuang’) alleles of *E4* gene have single-base deletions at different positions in exon 1 and exon 2, and *e4-SORE* (*Ty1*/*copia*-like retrotransposon) have a 6238-bp insertion in exon 1 [11,17,18]. Mutations at *E1*, *E3*, *E4* reduced photoperiod sensitivity of soybean to long daylength [16–18, 21–23], and various allele combinations of *E1*-*E4* underlie the flowering, maturity and ecological adaptation of soybean [24–26].

The SNPs and InDels that contribute to the phenotypic variations of crops are indispensable for the development of functional markers (FM) which are significant for germplasm evaluation and MAS. It has long been a challenge to visualize SNP genotyping with traditional molecular techniques while InDels can be identified easily. For genotyping of *E1*-*E4* genes, some allele specific markers, such as CAPS and dCAPS markers, which are based on PCR/gel and enzyme digestion, have been designed and used for genotyping of SNPs at *E1*-*E4* [15–18]. But the procedure of genotyping with these markers is complicated. In recent years, the KASP (Kompetitive Allele Specific PCR) genotyping technology has been applied to identify SNPs in some crops, such as wheat and rice [27,28]. KASP assays can achieve high throughput at low cost for genotyping, which greatly improves the efficiency of selection in breeding programs. Here, we developed KASP assays for high-throughput genotyping of *E1*-*E4* genes in soybean. We compared the effect of *E1*-*E4* on soybean flowering at different locations, and analyzed the associations between the allelic variations of four *E* genes and adaptation of Chinese soybean cultivars to diverse geographic regions and farming systems.

## Materials and methods

### Plant materials

A total of 308 soybean [*Glycine max* (L.) Merrill] cultivars released in 21 provinces of China were collected for genotyping in this study (S1 Table). The cultivars were classified into twelve MGs, including MG 0000, MG 000, MG 00, MG 0 and MGs I to VIII [29,30].

### Field trials and phenotyping

One hundred and ninety representative cultivars covering a wide range of MGs (S1 Table) were planted for phenotyping. Field trials were conducted in Beijing (40°13′ N, 116°33′ E) and Xinxiang (Henan province, China) (35°08′ N, 113°45′ E) in 2017. The cultivars were planted with two replicates in a randomized complete blocks design. All the cultivars were arranged in a 1.5 m row with 0.4 m apart between rows and a space of 0.1 m between adjacent plants. The field management followed local routine practices.

Five uniform plants of each line were selected for measurement of flowering time and maturity time. The flowering time was calculated as the number of days from emergence (VE) to beginning bloom (R1), and the maturity time was calculated as the number of days from emergence (VE) to beginning maturity (R7) [31]. It should be noted that only the date of R1 was recorded in Beijing because of the serious lodging of late-maturing plants at later stages of growth.

### Development of KASP assays

Since mutations at the maturity *E1*-*E4* loci made soybean reach flowering and maturity earlier [8–11,15–18], we sequenced cultivars from early-MGs to confirm the mutations or SNPs. In total, thirty cultivars from MGs 0000, 000, 00, 0, I-Ⅲ, were selected to Sanger sequence for SNP validation and KASP assays test (S2 Table). The genomic regions of *E1*, *E3* and *E4* were amplified for sequencing. The 10th exon of *E2* was sequenced to confirm the SNP resulting in the premature translation termination [9].

KASP assays were developed with the SNPs identified by sequencing. Two allele-specific forward primers were designed carrying unique tails- FAM (5′ GAAGGTGACCAAGTTCATGCT 3′) and HEX (5′ GAAGGTCGGAGTCAACGGATT 3′) respectively and with the targeted SNPs at the 3′ end, and a common primer was designed to pair with both forward primers. The InDels at *E3* and *E4* loci were genotyped with traditional InDel markers [18]. All primers for sequencing and genotyping are provided in S3 Table.

### Genotyping of *E1*-*E4*

The genomic DNA of the 308 cultivars was extracted using CTAB from fresh leaves. The KASP assays were tested with the above-mentioned thirty soybean cultivars (S2 Table). After initial testing, all 308 cultivars were genotyped with the KASP assays and InDel markers. In KASP assays, the primer mixture comprised ddH_2_O 46 μl, common primer 30 μl (100 μM), and each allele-specific forward primer 12 μl (100 μM). Assays were tested with 10 μl reactions (4.986 μl of 10–30 ng/μl genomic DNA, 5 μl of 2× KASP master mixture, and 0.014 μl of primer mixture). PCR cycling was performed using the following protocol: hot start at 95°C for 15 min, followed by 10 touchdown cycles (95°C for 20 s; touchdown from 65°C to 55°C with 1°C decrease per cycle for 60 s), followed by 30 additional cycles (95°C for 20 s; 57°C for 60 s). The fluorescent endpoint readings were performed on a real-time PCR system (ABI 7500). Traditional PCR was performed using EasyTaq DNA polymerase with 30 cycles at 94°C for 30 s, 58°C for 30 s and 72°C for 1 min, and the PCR products were separated on 1.0% agar gel [18].

### Statistical analysis

Analysis of variance (ANOVA) was performed based on four factors including *E* genes. Data analysis was conducted with the statistical software R (version 3.3.1) [32]].

## Results

### Genotyping of *E1*-*E4* in Chinese soybean cultivars with KASP assays

Six SNPs of *E1*-*E4* corresponding to *e1*-*as*, *e1*-*fs*, *e2*-*ns*, *e3*-*fs*, *e3*-*ns* and *e4*-*kes* alleles were detected by sequencing. Then, KASP assays (E1-SNP-as, E1-SNP-fs, E2-SNP-ns, E3-SNP-fs, E3-SNP-ns and E4-SNP-kes) were developed and tested with the thirty cultivars shown in S2 Table. The KASP assays of six SNPs produced results consistent with sequencing data and exhibited a good clustering of the homozygous alleles (S1 Fig).

All 308 cultivars were genotyped with six KASP assays and InDel markers. For the *E1* locus, two assays were performed for the genotyping of *e1-as* and *e1-fs*. The *e1-nl* allele was identified with PCR-electrophoresis, and amplification products were obtained for all cultivars except ‘Dongnong 41’. Among these cultivars, 0.6% carried the *e1-fs* allele, 31.8% carried the *e1-as* allele, and 67.2% carried the photoperiod-sensitive *E1* allele. For the *E2* locus, the *e2-ns* allele was identified in 83.1% of cultivars and the wild-type *E2* allele was mainly identified in late-maturing cultivars from the south. Five allelic variations of *E3* were identified, *e3-fs* and *e3-ns* were identified in 2.9% cultivars, *e3-tr* allele was identified in 31.2% cultivars, and 65.9% of cultivars carried the photoperiod-sensitive alleles *E3-Ha* or *E3-Mi*. Three alleles of *E4* were identified, the wild-type *E4* allele was identified in 96.8% of cultivars, *e4-SORE* and *e4-kes* were identified in 1.3% and 1.9% of the cultivars respectively.

Thus, a total of 21 allele combinations for *E1*-*E4* genes were identified across 308 cultivars (Table 1). Among them, *e1-as*/*e2-ns*/*e3-tr*/*E4*, *E1*/*e2-ns*/*E3-Mi*/*E4* and *E1*/*e2-ns*/*E3-Ha*/*E4* were dominant and accounted for 19.5%, 30.2% and 15.6% respectively.

**Table 1:** Allele combinations of four maturity *E* genes in Chinese soybean cultivars.

*E1*	*E2*	*E3*	*E4*	Number of cultivars
*e1-nl*	*e2-ns*	*e3-ns*	*e4-kes*	1
*e1-fs*	*e2-ns*	*e3-ns*	*e4-SORE*	1
*e1-fs*	*e2-ns*	*e3-tr*	*e4-kes*	1
*e1-as*	*e2-ns*	*e3-tr*	*e4-SORE*	3
*e1-as*	*e2-ns*	*e3-fs*	*e4-kes*	2
*e1-as*	*e2-ns*	*e3-tr*	*e4-kes*	2
*e1-as*	*E2*	*e3-tr*	*E4*	1
*E1*	*E2*	*e3-tr*	*E4*	1
*E1*	*e2-ns*	*e3-ns*	*E4*	1
*E1*	*E2*	*e3-ns*	*E4*	1
*e1-as*	*e2-ns*	*e3-fs*	*E4*	3
*e1-as*	*e2-ns*	*e3-tr*	*E4*	60
*e1-as*	*e2-ns*	*E3-Mi*	*E4*	8
*e1-as*	*e2-ns*	*E3-Ha*	*E4*	5
*e1-as*	*E2*	*E3-Mi*	*E4*	2
*e1-as*	*E2*	*E3-Ha*	*E4*	12
*E1*	*e2-ns*	*e3-tr*	*E4*	28
*E1*	*e2-ns*	*E3-Mi*	*E4*	93
*E1*	*e2-ns*	*E3-Ha*	*E4*	48
*E1*	*E2*	*E3-Mi*	*E4*	19
*E1*	*E2*	*E3-Ha*	*E4*	16

### Flowering and maturity time of soybean cultivars with different *E* allele combinations

**Fig 1:**
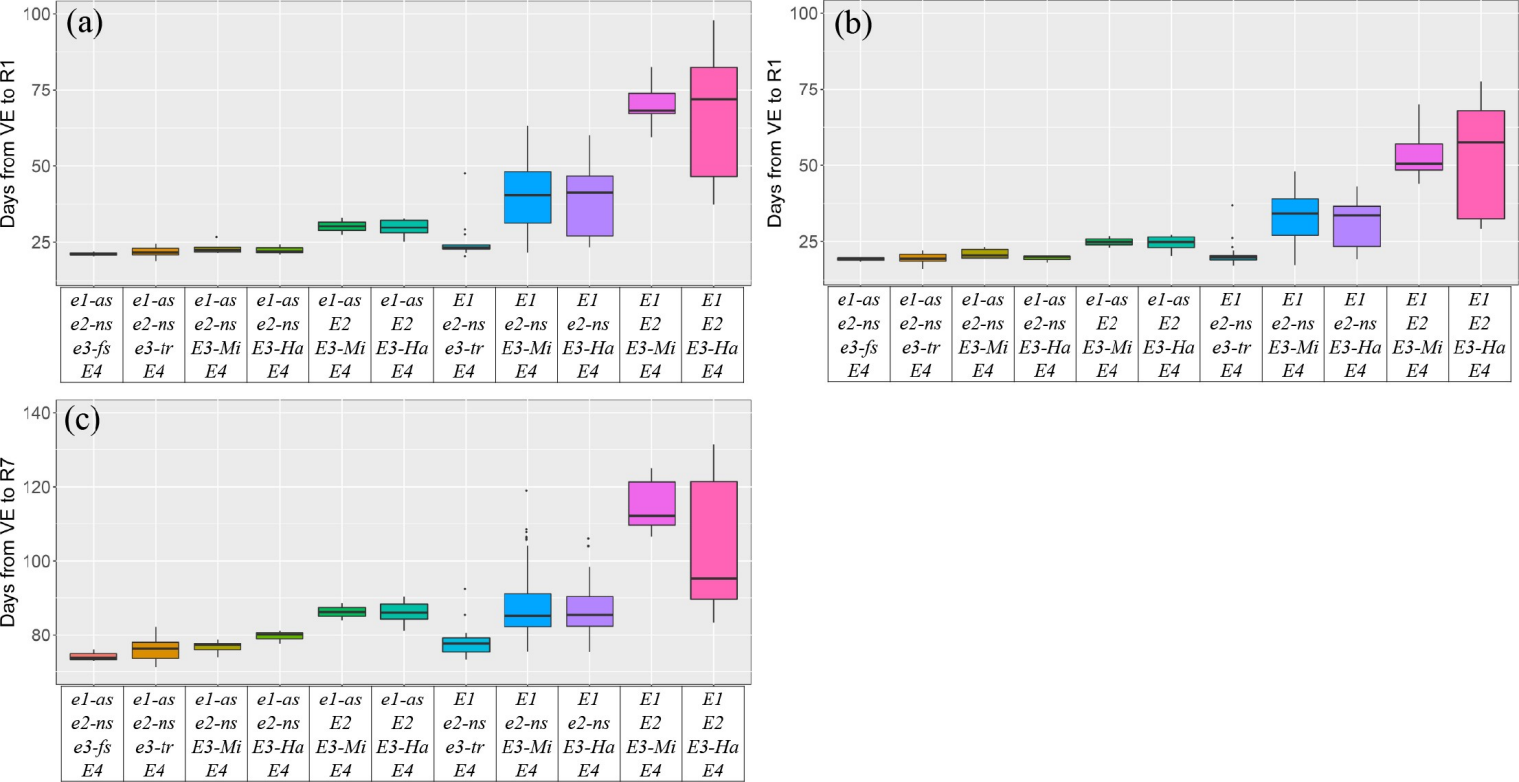
Flowering time (days from VE to R1) and maturity time (days from VE to R7) of different *E* allele combinations. (a) Flowering time of cultivars with different *E* allele combinations in Beijing. (b) Flowering time of cultivars with different *E* allele combinations in Xinxiang. (c) Maturity time of cultivars with different *E* allele combinations in Xinxiang. VE, emergence. R1, beginning bloom. R7, beginning maturity.

We identified two alleles of *E1* (*e1-as*, *E1*), two alleles of *E2* (*e2-ns*, *E2*), five alleles of *E3* (*e3-fs*, *e3-ns*, *e3-tr*, *E3-Ha*, *E3-Mi*) and one allele of *E4* (*E4*) in the 190 cultivars for phenotyping. The maturity time in Beijing dropped out of analysis because that R7 was not recorded at this location. Phenotypes of eleven allele combinations were analyzed based on the data collected in Beijing and Xinxiang (Fig 1, Table 2). By comparing the paired combinations *e1-as*/*e2-ns*/*E3-Mi*/*E4* and *e1-as*/*e2-ns*/*E3-Ha*/*E4*, *e1-as*/*E2*/*E3-Mi*/*E4* and *e1-as*/*E2*/*E3-Ha*/*E4*, *E1*/*e2-ns*/*E3-Mi*/*E4* and *E1*/*e2-ns*/*E3-Ha*/*E4* or *E1*/*E2*/*E3-Mi*/*E4* and *E1*/*E2*/*E3-Ha*/*E4*, there was little difference between *E3-Mi* and *E3-Ha* in terms of flowering and maturity time, and for this reason they were collectively referred to as *E3*.

**Table 2:** Flowering and maturity time of different *E* allele combinations.

Allele combination	Flowering time (d) Beijing	Flowering time (d) Xinxiang	Maturity time (d) Xinxiang
*e1-as*/*e2-ns*/*e3-fs*/*E4*	21.1±0.8	19.2±0.7	74.2±1.6
*e1-as*/*e2-ns*/*e3-tr*/*E4*	21.8±1.5	19.6±1.4	75.9±2.6
*e1-as*/*e2-ns*/*E3*-*Mi*/*E4*	23.1±2.1	21.0±1.7	76.6±1.8
*e1-as*/*e2-ns*/*E3*-*Ha*/*E4*	22.4±1.7	19.5±1.2	79.6±1.9
*e1-as*/*E2*/*E3*-*Mi*/*E4*	30.3±4.0	24.9±2.9	86.2±3.3
*e1-as*/*E2*/*E3*-*Ha*/*E4*	29.7±3.0	24.4±2.6	86.1±3.3
*E1*/*e2-ns*/*e3-tr*/*E4*	24.9±6.0	21.1±4.4	78.3±4.6
*E1*/*e2-ns*/*E3*-*Mi*/*E4*	40.6±11.7	32.9±8.3	88.3±9.6
*E1*/*e2-ns*/*E3*-*Ha*/*E4*	38.6±9.9	31.0±7.1	87.3±7.3
*E1*/*E2*/*E3*-*Mi*/*E4*	69.9±6.4	53.8±7.9	114.8±7.2
*E1*/*E2*/*E3*-*Ha*/*E4*	65.5±21.5	51.8±19.7	104.3±18.8

On the whole, the soybean cultivars reached flowering earlier in Xinxiang than in Beijing (Fig 1, Table 2). The cultivars carrying *e1-as*/*e2-ns*/*e3-fs*/*E4* reached flowering (19.2–21.1 days) and maturity (74.2 days) earliest (Fig 1, Table 2). There was little difference in flowering and maturity time among the allele combinations with mutations at both *E1* and *E2* loci, and cultivars with these genotypes reached flowering and maturity much earlier than others (Fig 1). In contrast, *e1-as*/*E2*/*E3*/*E4* and *E1*/*e2-ns*/*E3*/*E4* which contained the wild-type allele of *E1* or *E2* genes delayed flowering about five to nineteen days, and delayed maturity about twelve to fourteen days comparing to *e1-as*/*e2-ns*/*e3-fs*/*E4* (Fig 1, Table 2). Interestingly, the cultivars with *E1*/*e2-ns*/*e3*-*tr*/*E4*, though carrying the photoperiod-sensitive *E1* allele, flowered (21.1–24.9 days) and matured (78.3 days) very early, which was similar to the cultivars carrying mutations at both *E1* and *E2* loci (Fig 1, Table 2). No doubt, *E1*/*E2*/*E3*/*E4* delayed flowering and maturity most significantly, so that some cultivars carrying this allele combination didn’t reach maturity in Beijing or Xinxiang, Henan province.

Furthermore, we analyzed the effects of *E1*-*E4* on flowering and maturity of soybean by ANOVA. The variance of *E4* dropped out of analysis because no allelic variations were identified at *E4* locus across the cultivars for phenotyping. The ‘sum square’ of each *E* gene indicated the effects of the loci on flowering and maturity [18], the results showed that the effect of *E1* on flowering time was larger than *E2* in Beijing, but the effect of *E2* on flowering and maturity time was the largest in Xinxiang (Table 3).

**Table 3:** Analysis of Variance (ANOVA) of *E* genes for flowering and maturity time.

		Factor	D.f.	Mean square	F-value
**Flowering**	Beijing	*E1*	1	25928	269.63***
		*E2*	1	24957	259.54***
		*E3*	3	1543	16.05***
		REP	1	2	0.02
		*E1*:*E2*	1	7124	74.09***
		*E1*:*E3*	1	1769	18.40***
		*E2*:*E3*	1	710	7.38**
		Residuals	370	96	
	Xinxiang	*E1*	1	13019	216.87***
		*E2*	1	13319	221.88***
		*E3*	3	789	13.14***
		REP	1	0	0.00
		*E1*:*E2*	1	4342	72.32***
		*E1*:*E3*	1	965	16.08***
		*E2*:*E3*	1	429	7.15**
		Residuals	370	60	
**Maturity**	Xinxiang	*E1*	1	8937	139.68***
		*E2*	1	12112	189.30***
		*E3*	3	696	10.87***
		REP	1	54	0.84
		*E1*:*E2*	1	2111	33.00***
		*E1*:*E3*	1	534	8.34**
		*E2*:*E3*	1	373	5.83*
		Residuals	357	64	

LOC, location. REP, replication.

*Significant at P ≤ 0.05.

**Significant at P ≤ 0.01.

***Significant at P ≤ 0.001.

### Distribution of *E* genotypes in different maturity groups

**Fig 2:**
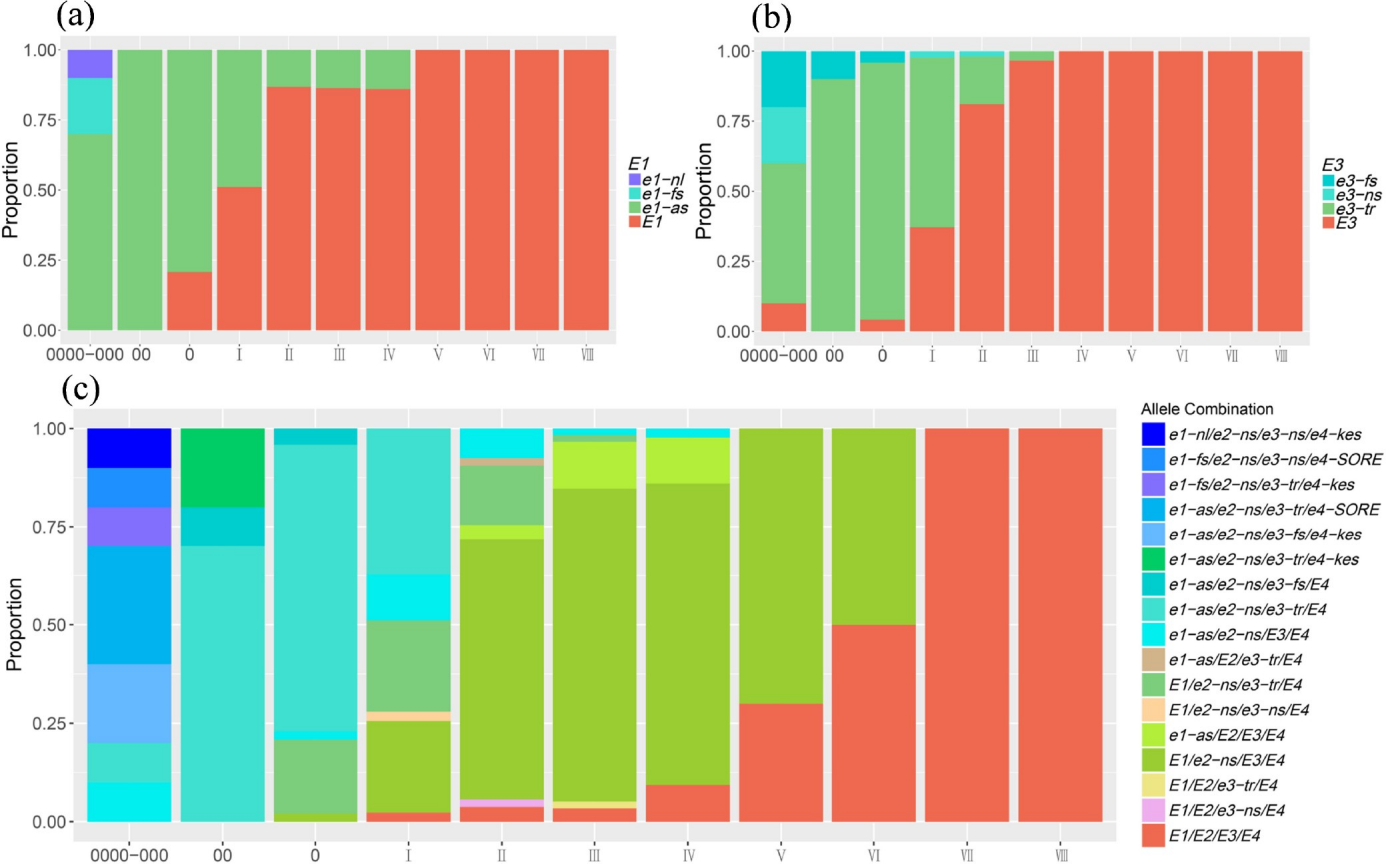
Distribution of *E* genotypes and their combinations in MGs. (a) The proportions of genotypes of *E1* in MGs. (b) The proportions of genotypes of *E3* in MGs. (c) The proportions of different *E* allele combinations in MGs.

The soybean cultivars carrying mutant alleles of *E1*-*E4* genes reached flowering and maturity earlier. The percentages of mutant alleles for *E1*, *E2*, *E3* and *E4* genes were 32.8%, 83.1%, 34.1%, and 3.2% in all 308 cultivars belonging to different MGs, respectively. Some infrequent alleles of *E1* (*e1-nl*, *e1-fs*), *E3* (*e3-fs*, *e3-ns*) and *E4* (*e4-SORE*, *e4-kes*) were identified in a few super early-maturing cultivars from MGs 0000–00 (Fig 2), indicating that the rare mutations may be associated with early maturity. The proportion of mutant alleles for these *E* genes became smaller from early-MGs to late-MGs (Fig 2). The mutant alleles *e1-as* and *e3-tr* were distributed in MGs 0000-Ⅳ and MGs 0000-Ⅱ respectively (Fig 2), while a majority of the Chinese cultivars carried *e2*-*ns* and *E4* alleles.

The combinations with mutations at all four *E* genes were mainly identified in MGs 0000–000. In this study, three major combinations *e1-as*/*e2-ns*/*e3-tr*/*E4*, *E1*/*e2-ns*/*E3*/*E4* and *E1*/*E2*/*E3*/*E4* accounted for 19.5%, 45.8% and 11.4% of all 308 cultivars, and played dominant roles in MGs 00-Ⅰ, MGs Ⅱ-Ⅴ and MGs Ⅵ-Ⅷ respectively (Fig 2). *E1*/*e2-ns*/*e3-tr*/*E4* accounted for 9.1% of 308 cultivars and was distributed in MGs 0-Ⅱ.

### Distribution of *E* genotypes in soybean cultivars from diverse geographic regions and farming systems in China

**Fig 3:**
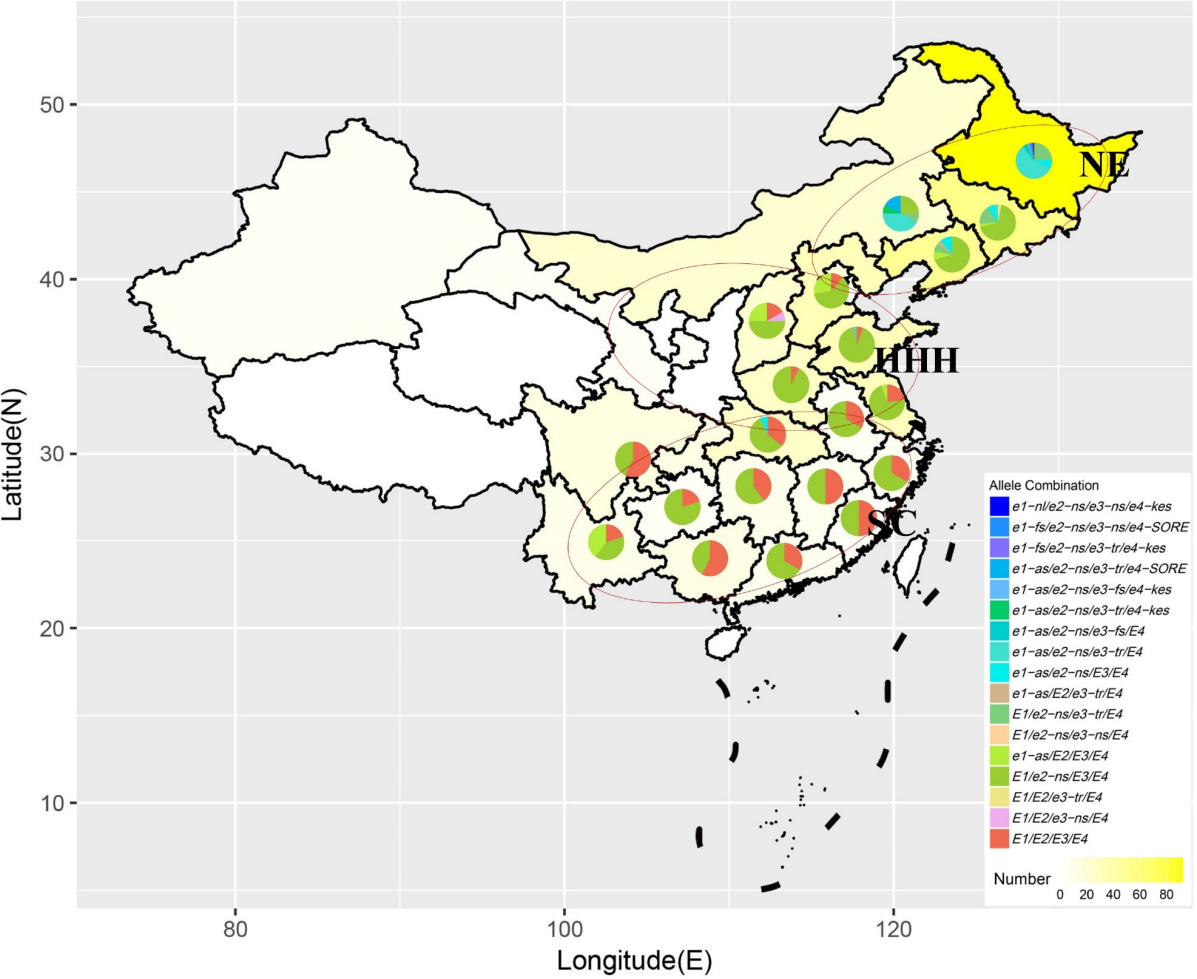
Geographic distribution of *E* allele combinations in China. Each pie chart indicates the proportional distribution of *E* allele combinations in its province. The intensity of yellow background represents the numbers of cultivars originated from each province. NE, Northeast China. HHH, Huang‐Huai‐Hai Rivers Valley Region. SC, South China.

Soybean is widely distributed in China and three major production regions are the Northeast, the Huang-Huai-Hai (HHH) Rivers Valley Region and the South (Fig 3) which differ considerably in terms of day-length and temperature. All the soybean cultivars in the Northeast are planted in the spring, thus the ecotype of the cultivars in this region is simplified as ‘North spring (N-sp)’. The soybean cultivars of the HHH region planted in the spring and summer are classified as ‘HHH spring (H-sp)’ and ‘HHH summer (H-su)’ respectively. In South China, the cultivars are assigned to ‘South spring (S-sp)’, ‘South summer (S-su)’ and ‘South autumn (S-au)’ based on their growing seasons.

Cultivars from twenty provinces were used to analyze the geographic distribution of *E* genotypes in China. The proportion of mutant alleles for these *E* genes decreased from north to south (Fig 3). Many mutant alleles or their combinations were identified in the Northeast. Most cultivars from the HHH region carried *E1*/*e2-ns*/*E3*/*E4*. The allele combination *E1*/*E2*/*E3*/*E4* was mainly distributed in the South.

Most mutations or mutant alleles were identified in N-sp cultivars which were planted in broad latitude ranging from 40°N to 53°N (Fig 3) and covered MGs 0000-IV (Table 4). *e1-as*/*e2-ns*/*e3-tr*/*E4* accounted for 36.6% of N-sp cultivars and played a dominant role in Northeast China. The cultivars which carried mutations at all four *E* loci were distributed in high-latitude (above 47° N) cold regions [3,29]. The cultivars from MGs 0-II adapted to the lower latitude in the Northeast, and also abundant allele combinations were identified in these MGs.

**Table 4:** *E* allele combinations and maturity groups of soybean cultivars of different ecotypes.

Ecotype	MG	*E* allele combinations
**N-sp**	0000	*e1*-*as*/*e2*-*ns*/*e3*-*tr*/*e4*-*SORE*
	000	*e1*-*nl*/*e2*-*ns*/*e3*-*ns*/*e4*-*kes*, *e1*-*fs*/*e2*-*ns*/*e3*-*ns*/*e4*-*SORE*, *e1*-*fs*/*e2*-*ns*/*e3*-*tr/e4*-*kes*,
		*e1*-*as*/*e2*-*ns*/*e3*-*fs*/*e4*-*kes*, *e1*-*as*/*e2*-*ns*/*e3*-*tr*/*e4*-*SORE*, *e1*-*as*/*e2*-*ns*/*e3*-*tr*/*E4*, *e1*-*as*/*e2*-*ns*/*E3*/*E4*
	00	*e1*-*as*/*e2*-*ns*/*e3*-*fs*/*E4*, *e1*-*as*/*e2*-*ns*/*e3*-*tr*/*e4*-*kes*, *e1*-*as*/*e2*-*ns*/*e3*-*tr*/*E4*
	0	*e1*-*as*/*e2*-*ns*/*e3*-*fs*/*E4*, *e1*-*as*/*e2*-*ns*/*e3*-*tr*/*E4*, *e1*-*as*/*e2*-*ns*/*E3*/*E4*, *E1*/*e2*-*ns*/*e3*-*tr*/*E4*, *E1*/*e2*-*ns*/*E3*/*E4*
	Ⅰ	*e1*-*as*/*e2*-*ns*/*e3*-*tr*/*E4*, *e1*-*as*/*e2*-*ns*/*E3*/*E4*, *E1*/*e2*-*ns*/*e3*-*tr*/*E4*, *E1*/*e2*-*ns*/*e3*-*ns*/*E4*, *E1*/*e2*-*ns*/*E3*/*E4*
	Ⅱ	*e1*-*as*/*e2*-*ns*/*E3*/*E4*, *E1*/*e2*-*ns*/*e3*-*tr*/*E4*, *e1*-*as*/*E2*/*E3*/*E4*, *e1*-*as*/*E2*/*e3*-*tr*/*E4*, *E1*/*e2*-*ns*/*E3*/*E4*
	Ⅲ	*e1*-*as*/*e2*-*ns*/*E3*/*E4*, *e1*-*as*/*E2*/*E3*/*E4*, *E1*/*e2*-*ns*/*E3*/*E4*, *E1*/*E2*/*e3*-*tr*/*E4*
	Ⅳ	*e1*-*as*/*e2*-*ns*/*E3*/*E4*, *E1*/*e2*-*ns*/*E3*/*E4*
**H-sp**	Ⅱ	*E1*/*e2*-*ns*/*E3*/*E4*, *E1*/*E2*/*e3*-*ns*/*E4*, *E1*/*E2*/*E3*/*E4*
	Ⅲ	*e1*-*as*/*E2*/*E3*/*E4*, *E1*/*e2*-*ns*/*E3*/*E4*
	Ⅳ	*e1*-*as*/*E2*/*E3*/*E4*, *E1*/*e2*-*ns*/*E3*/*E4*, *E1*/*E2*/*E3*/*E4*
	Ⅴ	*E1*/*E2*/*E3*/*E4*
**H-su**	Ⅰ	*E1*/*e2*-*ns*/*E3*/*E4*
	Ⅱ	*E1*/*e2*-*ns*/*E3*/*E4*
	Ⅲ	*E1*/*e2*-*ns*/*e3*-*tr*/*E4*, *e1*-*as*/*E2*/*E3*/*E4*, *E1*/*e2*-*ns*/*E3*/*E4*, *E1*/*E2*/*E3*/*E4*
	Ⅳ	*e1*-*as*/*E2*/*E3*/*E4*, *E1*/*e2*-*ns*/*E3*/*E4*, *E1*/*E2*/*E3*/*E4*
	Ⅴ	*E1*/*e2*-*ns*/*E3*/*E4*
**S-sp**	Ⅰ	*e1*-*as*/*e2*-*ns*/*E3*/*E4*, *E1*/*e2*-*ns*/*E3*/*E4*, *E1*/*E2*/*E3*/*E4*
	Ⅱ	*E1*/*e2*-*ns*/*E3*/*E4*, *E1*/*E2*/*E3*/*E4*
	Ⅲ	*e1*-*as*/*E2*/*E3*/*E4*, *E1*/*e2*-*ns*/*E3*/*E4*, *E1*/*E2*/*E3*/*E4*
	Ⅳ	*e1*-*as*/*E2*/*E3*/*E4*, *E1*/*e2*-*ns*/*E3*/*E4*
	Ⅴ	*E1*/*E2*/*E3*/*E4*
	Ⅵ	*E1*/*e2*-*ns*/*E3*/*E4*
**S-su**	Ⅳ	*E1*/*e2*-*ns*/*E3*/*E4*
	Ⅴ	*E1*/*e2*-*ns*/*E3*/*E4*
	Ⅵ	*E1*/*e2*-*ns*/*E3*/*E4*, *E1*/*E2*/*E3*/*E4*
	Ⅶ	*E1*/*E2*/*E3*/*E4*
	Ⅷ	*E1*/*E2*/*E3*/*E4*
**S-au**	Ⅷ	*E1*/*E2*/*E3*/*E4*

N-sp, North spring. H-sp, Huang-Huai-Hai spring. H-su, Huang-Huai-Hai summer. S-sp, South spring. S-su, South summer. S-au, South autumn.

In the HHH region, *E1*/*e2*-*ns*/*E3*/*E4* accounted for 76.0% of HHH cultivars while *e1-as*/*E2*/*E3*/*E4* were mainly identified in H-sp soybean cultivars. Some cultivars carrying *E1*/*E2*/*E3*/*E4* were identified in this region. However, the cultivars carrying *E1*/*E2*/*E3*/*E4* in the HHH region reached maturity earlier than those from the South region, which resulted in a large variation range of flowering and maturity time of *E1*/*E2*/*E3*/*E4* (Fig 1).

In the South, *E1*/*e2*-*ns*/*E3*/*E4* made up a large proportion of S-sp soybean cultivars (MG I to Ⅵ, Table 4), while *E1*/*E2*/*E3*/*E4* played dominant roles in S-su and S-au cultivars (MG IV to VIII, Table 4). Wild-type alleles were mostly identified in the soybean cultivars from the South, but some allele combinations associated with early maturity, such as *e1-as*/*e2-ns*/*E3*/*E4*, were also identified in the early-maturing cultivars (MG I) from this region (Table 4, S1 Table). The early-maturing cultivars from South China, such as ‘Edou 8’ (*e1-as*/*e2-ns*/*E3*/*E4*), ‘Taixingheidou’ (*e1-as*/*E2*/*E3*/*E4*) and ‘Edou 4’(*E1*/*E2*/*E3*/*E4*), were S-sp cultivars which were relatively photoperiod-insensitive.

## Discussion

### Alleles of *E1-E4* were identified to different proportions in Chinese soybean

Genetic and molecular analyses have revealed that various mutations of *E1-E4* genes had effects on flowering and maturity of soybean [15–18,24–26]. Mutations at four *E* loci influence flowering and maturity at different levels [18,25,26]. The effect of some mutations in the 5′ upstream regions or introns on flowering of soybean is still unclear [18]. Some alleles (*e4-kam*, *e4-tsu*, *e4-oto* and *e3-Mo*) identified previously in Japanese, Russian and a few Chinese cultivars [16,17] were not detected in this study, implying these alleles might play minor roles in maturity time improvement of soybean breeding in China. Some alleles were not detected maybe due to the deficiency of cultivars for sequencing, therefore more cultivars with different origins are in need for further identification of natural variations.

Multiple allelic variations of *E1* and *E3* were identified across the Chinese soybean cultivars used in the study. *e1-as* and *e3-tr* were identified in numerous cultivars, indicating that they contributed to regulate maturity of Chinese soybean greatly. In contrast, the photoperiod-insensitive alleles of *E4* were identified only in several super early cultivars belonging to MGs 0000–000. It implied that the *E4* locus might have a small contribution to flowering and maturity of Chinese soybean. For the *E2* gene, a majority of the Chinese soybean cultivars possessed the *e2-ns* allele, which was consistent with previous studies [24,25]. This may be because *E2* locus was not responsible for the photoperiod sensitivity but contributed to the expansion of cultivated soybean [33,34]. Understanding the natural variations of the loci controlling flowering and maturing is very important for MAS and molecular design breeding of soybean in the future.

### *E1*-*E4* played different roles in controlling flowering and maturity of soybean

Each of *E1*-*E4* genes performs a different function and influences photoperiod sensitivity and flowering time of soybean at a different extent. ANOVA results showed that *E1* had larger effect on flowering time than *E2* in Beijing (40°13′ N, 116°33′ E), but *E1* had smaller effect than *E2* in Xinxiang (35°08′ N, 113°45′ E), which may be due to the longer daylength of Beijing than Xinxiang. Because *E1* was induced by long daylength to suppress flowering of soybean [8], then it maybe had larger effect under the longer daylength of Beijing. This may be also the reason that soybean reached flowering earlier in Xinxiang than in Beijing.

In the previous studies, *E1* was proved to have the largest effect on flowering in soybean [18,35,36], *E2* had an obvious effect on maturity under short-day conditions [37]. In this study, *E2* showed large effect on flowering and maturity of soybean. Previous reports also suggested that *E2* exhibited pleiotropy across different traits including flowering and maturity in soybean and also played an important role in regulating the traits related to yield and seed quality [38–40]. Further studies are thus needed to reveal the molecular basis of the *E2* gene in the control of flowering and maturity.

In the background of *e2-ns*/*e3-tr*/*E4*, the cultivars carrying the photoperiod-sensitive *E1* allele reached flowering and maturity very early, which was similar to the cultivars carrying *e1*-*as* (Fig 1). However, in the background of *e2-ns*/*E3*/*E4*, *E1* delayed flowering and maturity in comparison with *e1-as*. Thus, *E1* hardly repressed flowering in the background of *E3* inactivation, suggesting that the inhibition of *E1* was under the control of *PHYA* gene *E3* [8].

The various allele combinations of *E1*-*E4* gave rise to the diversity of flowering and maturity time of soybean cultivars. Cultivars carrying the same allele combinations may vary in flowering and maturity time, suggesting that other genes (e.g. *E5*-*E10*, *J*) or other unknown mutations of four *E* loci are involved in the control of flowering [12–14,18,41–44]. Gene-environment interaction may be another important factor that affects flowering and maturity time. Further studies should be conducted to identify other genes controlling flowering and maturity time, and to comprehensively analyze interactions among these genes and genotype × environmental interaction.

### Allele combinations of *E1*-*E4* affected adaptation of soybean to diverse geographic regions and farming systems

Mutations of *E1*-*E4* genes reduced the photoperiod sensitivity and shorten the growth period of soybean cultivars [16–18,21–23], which is essential for soybean adaptation to long daylength or short growing season. Various combinations of mutations at four maturity *E* loci made cultivars adapt to different latitude environments and farming systems.

In the Northeast, the N-sp cultivars carrying recessive alleles of four *E* genes reached maturity early and belonged to early-MGs. Multiple mutations of four *E* genes made these soybean cultivars photoperiod-insensitive and able to adapt to the long-day conditions during the growing season of high latitudes in China. In particular, cultivars with mutations at all four *E* genes were distributed in MGs 0000–00 and matured very early, which is essential for adapting to high-latitude cold regions. Interestingly, these cultivars with mutations at all four *E* loci were not uniform in flowering and maturity time, indicating that other novel loci besides *E1*-*E4* are involved in the control of flowering and maturity in these early cultivars. In the HHH region, most of the cultivars belonged to medium-MGs. A majority of H-su cultivars carried *E1*/*e2-ns*/*E3*/*E4*, while *e1-as*/*E2*/*E3*/*E4* were mainly identified in ‘HHH spring’ cultivars which were photoperiod-insensitive compared to the summer cultivars, indicating that *e1-as*/*E2*/*E3*/*E4* may be more insensitive to long-day than *E1*/*e2-ns*/*E3*/*E4*. In the South, the photoperiod-sensitive allele combination *E1*/*E2*/*E3*/*E4* was mainly identified in S-su and S-au belonging to late-MGs and contributed to the adaptation of soybean to the short-day conditions. The photoperiod-insensitive S-sp cultivars reached flowering and maturity earlier than S-su and S-au cultivars. Some of the S-sp cultivars belonged to early-MGs Ⅰ-Ⅱ, among them some carried early-flowering allele combinations, such as *e1-as*/*e2-ns*/*E3*/*E4*, while others carried late-flowering allele combinations *E1*/*E2*/*E3*/*E4* (S1 Table). The mechanism by which some S-sp cultivars carrying *E1*/*E2*/*E3*/*E4* flower and mature earlier than other cultivars carrying same allele combination need to be further studied.

In this study, three allele combinations *e1-as*/*e2-ns*/*e3-tr*/*E4*, *E1*/*e2-ns*/*E3*/*E4* and *E1*/*E2*/*E3*/*E4* played important roles in the Northeast, HHH and South regions, respectively. The allele combination *E1*/*e2-ns*/*E3*/*E4* was identified in 45.8% cultivars of MGs Ⅰ-Ⅵ, reflecting its universality in Chinese soybean [25].

## Conclusion

Various combinations of mutations of *E1*-*E4* genes gave rise to the diversity of flowering and maturity time of soybean and enabled the cultivars adapt to different ecological regions and multiple cropping systems in China. The allele combinations *e1-as*/*e2-ns*/*e3-tr*/*E4*, *E1*/*e2-ns*/*E3*/*E4* and *E1*/*E2*/*E3*/*E4* played important roles in the adaptation of the soybean cultivars to the Northeast, HHH Region and the South in China respectively. The KASP assays developed in this study will facilitate the germplasm characterization and MAS of maturity in soybean breeding programs.

## Supporting information

S1 Fig **Scatter plots of KASP genotyping showing clustering of cultivars on the X- (HEX) and Y- (FAM) axes.** (a) KASP assay of E1-SNP-as showing C (*e1-as*) on FAM and G on HEX clusters. (b) KASP assay of E1-SNP-fs showing A on FAM and A-deletion (*e1-fs*) on HEX clusters. (c) KASP assay of E2-SNP-ns showing A on FAM and T (*e2-ns*) on HEX clusters. (d) KASP assay of E3-SNP-fs showing T on FAM and Ins T (*e3-fs*) on HEX clusters. (e) KASP assay of E3-SNP-ns showing C on FAM and T (*e3-ns*) on HEX clusters. (f) KASP assay of E4-SNP-kes showing A on FAM and A-deletion (*e4-kes*) on HEX clusters.
(TIF)

S1 Table Cultivars for phenotyping and distribution analysis. (XLSX)

S2 Table SNPs and genotypes of *E1*-*E4* in thirty soybean cultivars for sequencing. ^1^ Numbers represent the position of SNPs from the start codon in genomic region. ‘-’, Deletion. ‘Ins’, Insertion.
(XLSX)

S3 Table Primers for cloning and genotyping. (XLSX)
