# Supplementary figures and images for: Allele combinations of maturity genes E1-E4 affect adaptation of soybean to diverse geographic regions and farming systems in China
Source: PLoS One. 2020 Jul 6;15(7):e0235397. doi: 10.1371/journal.pone.0235397 (PMC7337298; doi:10.1371/journal.pone.0235397)

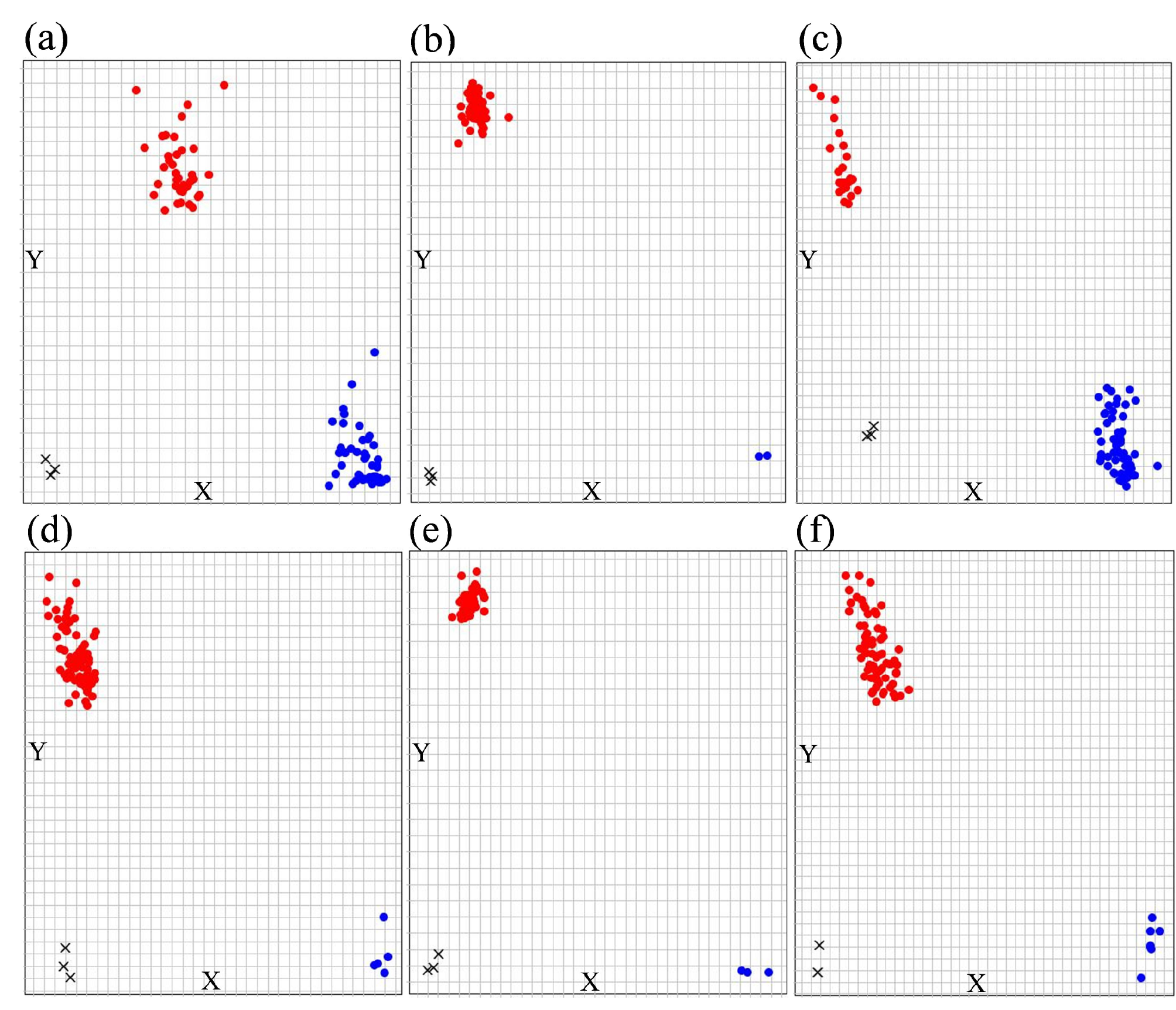

Supplement: S1 Fig — Scatter plots of KASP genotyping showing clustering of cultivars on the X- (HEX) and Y- (FAM) axes. (a) KASP assay of E1-SNP-as showing C (e1-as) on FAM and G on HEX clusters. (b) KASP assay of E1-SNP-fs showing A on FAM and A-deletion (e1-fs) on HEX clusters. (c) KASP assay of E2-SNP-ns showing A on FAM and T (e2-ns) on HEX clusters. (d) KASP assay of E3-SNP-fs showing T on FAM and Ins T (e3-fs) on HEX clusters. (e) KASP assay of E3-SNP-ns showing C on FAM and T (e3-ns) on HEX clusters. (f) KASP assay of E4-SNP-kes showing A on FAM and A-deletion (e4-kes) on HEX clusters. (TIF) [file pone.0235397.s001.tif]
